# The reciprocal relationship between physical activity and prosocial behavior among rural left-behind children: a cross-lagged mediation analysis of psychological resilience

**DOI:** 10.3389/fpubh.2025.1690603

**Published:** 2025-12-03

**Authors:** Dawu Huang, Xin Xiong, Peiyi Wang, Juan Xu

**Affiliations:** 1 School of Physical Education, Jiangxi Normal University, Nanchang, China; 2 Minnan University of Science and Technology, Fujian, China; 3 Jiangxi Normal University, Nanchang, China; 4 Department of Psychological Science, University of Texas, Rio Grande Valley, Edinburg, TX, United States; 5 Chongqing City Hospital of Traditional Chinese Medicine, Chongqing, China

**Keywords:** physical activity, prosocial behavior, psychological resilience, rural left-behind children, longitudinal mediation

## Abstract

**Introduction:**

Although physical activity benefits youth development, little is known about how it is related to prosocial behavior over time, especially among structurally disadvantaged groups such as rural left-behind children in China. This longitudinal study examined the bidirectional relationship between physical activity and prosocial behavior and the mediating role of psychological resilience.

**Methods:**

A total of 612 children (Grades 5–6; approximately 10–12 years old; 50.5% male) from three schools in Ji’an City, Jiangxi Province, completed measures of physical activity, psychological resilience, and prosocial behavior at three time points over 6 months.

**Results:**

Crosslagged mediation structural equation modeling revealed reciprocal pathways: Time 1 physical activity was related to Time 3 prosocial behavior, and vice versa. In both directions, psychological resilience at Time 2 significantly mediated these associations (indirect effects = 0.07 and 0.04, respectively; *p*s < 0.001). These findings underscore psychological resilience as a key developmental mechanism linking physical and social functioning.

**Discussion:**

The results highlight the potential for integrated school-based interventions that promote physical activity to enhance psychological resilience and social functioning in vulnerable youth populations, particularly rural left-behind children.

## Introduction

Urbanization represents one of the most significant global demographic trends, profoundly reshaping social, economic, and cultural structures worldwide (1). Particularly in China, rapid urbanization has driven unprecedented rural-to-urban migration, resulting in psychosocial challenges (2). Among these challenges is the rising number of rural left-behind children, those whose parents migrate to urban areas for employment opportunities, leaving children behind in rural settings (3). Official statistics indicate that there are approximately 69 million rural left-behind children in China (4) who are subjective to severe emotional distress, delays in psychological and social development, and impaired social adaptability partly due to prolonged parental absence and limited access to support (5). The economic cost associated with managing the consequences of parental migration, including educational support, mental health care, and social services, creates long-term socioeconomic burdens of the country (6). Given the global prevalence of migration and similar challenges in other regions (e.g., child migrants or sent-away children), researchers worldwide should prioritize the psychosocial health of these individuals. Specifically, it is critical to prospectively examine the associations between physical activity, considered as a potential intervention target, and psychosocial outcomes among rural left-behind children (7).

One critical psychosocial concern among rural left-behind children is their reduced display of prosocial behavior (8). Prosocial behavior refers to positive interactions beneficial to others and social harmony and is an essential indicator of mental health and social stability (9). With the severance of emotional ties, left-behind children often experience negative emotions such as stress, anxiety, sadness, depression, and self-isolation, leading to low levels of prosocial behavior and poor social adjustment (10). Neurological research revealed that parental absence significantly impacts brain regions associated with emotional and social functioning. For example, prolonged parental separation correlates with increased amygdala volume (11, 12), a brain structure essential for emotional processing and social interactions, potentially reducing prosocial behaviors. Additionally, adverse experiences disrupt neural synchrony in lateral prefrontal cortex, an area crucial for emotion regulation and social cognition (13). In the same vein, positive parent–child interactions play a critical role in developing neural circuits involved in empathy and prosocial behavior (14). A meta-analysis examining parenting behaviors found significant positive correlations between nurturing parenting practices (i.e., child–mother attachment security) and children’s prosocial behaviors (15).

Given these socio-emotional vulnerabilities, identifying accessible and developmentally appropriate strategies is critical to support prosocial development among rural left-behind children. One promising pathway may lie in the role of physical activity. On one hand, physical activity can promote prosocial behavior by fostering healthy self-perceptions and enhancing emotional intelligence (16–18). For example, a positive campus sports environment has been associated with higher levels of children’s subjective well-being through improved peer relationships and positive emotions (19). These experiences lay the foundation for the development of prosocial tendencies in children. Regular participation in physical activity has also been associated with increased activation in the prefrontal cortex, a region critical for social decision-making and emotional regulation (20, 21). A meta-analytical review among children and adolescent further shows that playing sports can improve their prosocial behaviors (22). Interestingly, emerging evidence suggests that this relationship may be bidirectional: children who display higher levels of prosocial behavior may also engage more frequently in physical activity, particularly in cooperative or socially interactive contexts (23). Such children with high prosocial behaviors may actively seek social participation opportunities that encourage involvement in group-based exercise or sports. Some studies among older adults also showed that incorporating prosocial behaviors can promote physical activity (24). However, due to the cross-sectional designs of most studies, the directionality and temporal dynamics of this association has not been established. Therefore, longitudinal evidence is needed to clarify the bidirectional associations between physical activity and prosocial behavior and to elucidate their underlying mechanisms.

Psychological resilience may serve as a mediating mechanism in both directions of the relationship between physical activity and prosocial behavior. Resilience theory (25) posits that individuals with higher psychological resilience are better able to maintain adaptive functioning in the face of adversity, enhancing their capacity to recover from challenges and sustain positive social and health-related behaviors (26). On one hand, physical activity may be associated with greater psychological resilience, which in turn is linked to higher levels of prosocial behavior. Theoretically, physical activities can fulfill psychological needs such as competence, autonomy, and relatedness, which are foundational to building psychological resilience (27, 28). This is supported by empirical and meta-analytical review evidence (29), showing that physical activity is positively associated with psychological resilience among children and adolescents (30–32), by providing structured opportunities to overcome challenges, develop self-regulatory skills, and build coping strategies (33–35). Psychological resilience, in turn, has been identified as a predictor of prosocial behaviors by empirical research (36, 37), likely due to greater empathy and cooperative problem-solving (38). However, a small body of evidence also suggests that prosocial behavior may be associated with greater psychological resilience. For example, a quantitative study among single mothers found that prosocial behaviors predicted higher resilience by fostering a psychological sense of community and promoting positive personal outcomes (39). In the context of sports, resilience-enhancement programs have been implemented to improve athletes’ persistence and performance (40, 41), suggesting that resilience may serve as a predictor of sustained physical activity. However, a notable research gap persists concerning longitudinal studies explicitly investigating psychological resilience as a mediator in the potential bidirectional association between physical activity and prosocial behavior among rural left-behind children. This is particularly important because the development of effective interventions requires a clear understanding of not only the directionality of these relationships but also the underlying psychological mechanisms.

**Figure 1:**
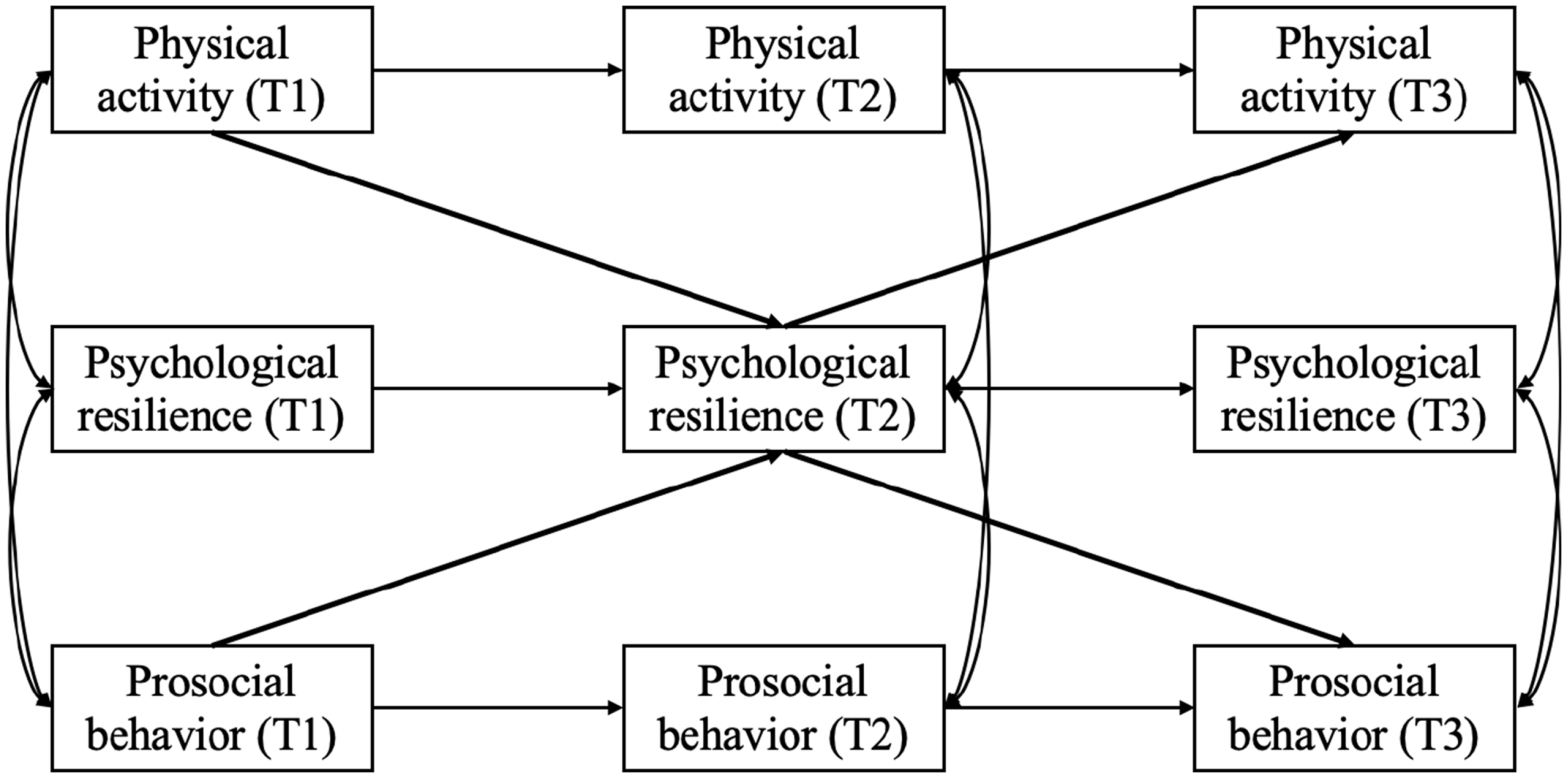
Proposal study model.

The present study aimed to fill this gap by using a longitudinal design with three measurement points to examine whether psychological resilience mediates the relationship between physical activity and prosocial behavior, in both directions, over time among rural left-behind children in China (Figure 1). It was hypothesized that: (1) physical activity is positively related to psychological resilience, which in turn linked to prosocial behavior; (2) prosocial behavior is positively linked to psychological resilience, which in turn, related to physical activity; and (3) psychological resilience serves as a longitudinal mediator in both pathways.

## Method

### Participants

A convenience sampling method was used. Fifth- and sixth-grade students from three rural primary schools in Ji’an City voluntarily participated this study. Ji’an City’s rural areas are primarily sustained by traditional agriculture, and average household income remains below the national rural average. Many working-age adults migrate to urban centers for employment, leaving a considerable number of children in the care of relatives. Local schools have limited educational resources and less qualified teaching staff compared to urban schools. A convenience sampling method was adopted due to the wide geographic distribution of rural schools and the need for cooperation from both school administrators and parents. This approach ensured feasibility and facilitated participant access under real-world field conditions.

Eligibility was determined using a screening item that explicitly asked whether both parents had been employed away from home for more than 6 months, or whether one parent had been employed away from home while the other lacked caregiving capacity. Participants who responded “Yes” to this item were classified as left-behind children and included in the study.

The final sample consisted of 612 participants who completed all three assessments (retention rate: 85%). Among them, 309 participants (50.5%) were male and 303 (49.5%) were female; 338 (55.2%) were in fifth grade and 274 (44.8%) were in sixth grade, corresponding to an approximate age range of 10 to 12 years based on the standard Chinese education system. Participant demographic information is presented in Table 1. The initial pool consisted of 716 valid questionnaires collected in October 2023 (T1), 699 in January 2024 (T2), and 711 in April 2024 (T3). Cases were excluded if they met any of the following criteria: excessive missing data (i.e., more than two-thirds of items unanswered), inconsistencies between positively and negatively worded items, patterned or uniform responses, or missing identification information. Participant attrition was primarily due to school transfers, graduation, or absences. Attrition analyses indicated no significant differences at T1 between participants who dropped out and those who completed all three waves in physical activity (*t* = −0.017, *p* > 0.05), prosocial behavior (*t* = 0.994, *p* > 0.05), or psychological resilience (*t* = −1.645, *p* > 0.05), suggesting that attrition was not systematic.

**Table 1:** Participant demographic information (*N* = 612).

Variable	Category	*n*	Frequency
Sex	Boy	309	50.5%
	Girl	303	49.5%
Grade	Fifth	338	55.2%
	Sixth	274	44.8%
Only Child	Yes	52	8.5%
	No	560	91.5%

### Procedure

This study adopted a three-wave longitudinal design (T1, T2, and T3) conducted over a six-month period, with three-month intervals between waves. All procedures were approved by the Institutional Review Board (IRB-JXNU-PEC-20230901). Through three collaborating public schools in Ji’an City, Jiangxi Province, written informed consent forms detailing the nature of this longitudinal study were distributed to the parents or legal guardians of students in Grades 5 and 6. A total of 612 students were included in the final sample after obtaining parental consent. Data were collected at three time points: October 2023 (T1), January 2024 (T2), and April 2024 (T3).

Before each data collection, trained research staff visited the classrooms and explained the study purpose, procedures, and confidentiality protections in age-appropriate language. Students were informed that participation was entirely voluntary and that they could skip any question or withdraw at any time without penalty. After confirming comprehension, the researchers obtained verbal assent from each student before administering the questionnaires.

Participants completed self-report questionnaires assessing physical activity, psychological resilience, and prosocial behavior in a classroom setting, which took approximately 10–15 min to complete. Physical activity was assessed through self-report to ensure a culturally appropriate, feasible, and minimally intrusive method for large-scale, longitudinal data collection among rural left-behind children. To ensure comprehension, all instruments were validated among Chinese children and reviewed for age-appropriate language. A pilot test with 30 students of similar age from non-participating schools was conducted to evaluate comprehension and optimize wording clarity.

During data collection, trained research assistants read standardized instructions aloud, provided sample items to demonstrate the response format, and closely monitored the session. When a student appeared confused, assistants provided neutral clarifications without suggesting answers. For example, when a student did not understand the resilience item “I can recover quickly from unpleasant experiences,” the assistant clarified, “This question is asking whether you can make yourself feel better quickly after something makes you unhappy.”

## Measures

### Physical activity questionnaire

Physical activity was measured by the Chinese version of the Physical Activity Rating Scale [(42); 4 items] and three additional questions assessing the psychological perception of physical activity (43). The original PARS evaluates behavioral dimensions of physical activity, including intensity, average duration per session, frequency, and subjective feelings after exercise. The three additional items assess emotional preference (e.g., “Do you enjoy physical activity?”), perceived benefits (e.g., “Do you believe regular exercise is beneficial for your health?”), and self-motivation (e.g., “Do you engage in physical activity without being prompted by others?”). All items were rated on a 5-point Likert-type scale, with higher scores indicating greater engagement in and more positive perceptions of physical activity. For example, exercise intensity was assessed on a scale ranging from 1 (*light activities such as walking*) to 5 (*vigorous and sustained activities such as competitive swimming*). Frequency ranged from 1 (*less than once per month*) to 5 (*almost daily*). Higher total scores indicate greater physical activity engagement. Because both the behavioral and psychological perception components are theoretically relevant to children’s overall engagement in physical activity and have been integrated in prior research (43), the seven items were combined into a single composite score.

This integration was supported by satisfactory internal consistency across all three time points (Cronbach’s *α* = 0.84 at T1, 0.78 at T2, and 0.78 at T3) and strong construct validity as indicated by confirmatory factor analyses (T1: *χ*^2^/df = 1.26, CFI = 0.99, TLI = 0.99, RMSEA = 0.02; T2: *χ*^2^/df = 2.37, CFI = 0.98, TLI = 0.97, RMSEA = 0.05; T3: *χ*^2^/df = 1.81, CFI = 0.99, TLI = 0.98, RMSEA = 0.04). Kaiser–Meyer–Olkin (KMO) values indicated good sampling adequacy (T1 = 0.88; T2 = 0.85; T3 = 0.85), and Bartlett’s test of sphericity was significant across all time points (all *p* < 0.001).

### Adolescent prosocial behavior scale

Prosocial behavior was assessed using the Chinese version of the Adolescent Prosocial Tendencies Scale (44, 45). The scale consists of 26 items measuring six types of prosocial tendencies: anonymous, public (overt), altruistic, emotional, compliant, and urgent. Participants rated each item on a 5-point Likert scale ranging from 1 (*completely untrue*) to 5 (*completely true*), with higher scores indicating greater prosocial tendencies. An example item is: “I often help others, even when I do not receive any benefit in return.”

Cronbach’s alpha coefficients demonstrated high reliability at all measurement points: 0.92 (T1), 0.93 (T2), and 0.93 (T3). Confirmatory factor analyses showed good structural validity (T1: *χ*^2^/df = 2.20, CFI = 0.94, TLI = 0.93, RMSEA = 0.04; T2: *χ*^2^/df = 3.00, CFI = 0.91, TLI = 0.90, RMSEA = 0.06; T3: *χ*^2^/df = 1.99, CFI = 0.95, TLI = 0.94, RMSEA = 0.04). KMO values confirmed sampling adequacy (T1 = 0.95; T2 = 0.94; T3 = 0.95), and Bartlett’s test of sphericity was significant across measurement points (all *p* < 0.001).

### Psychological resilience scale

Psychological resilience was evaluated using the Resilience Scale for Adolescents (46). The scale consists of 27 items across five subscales: goal focus, emotional control, positive cognition, family support, and interpersonal assistance. Items were rated on a 5-point Likert scale ranging from 1 (*completely untrue*) to 5 (*completely true*). After reverse coding specified items, higher scores reflected greater psychological resilience. An example item is: (“I believe adversity can be motivating for individuals”).

Cronbach’s alpha indicated acceptable internal consistency: 0.83 (T1), 0.87 (T2), and 0.87 (T3). Confirmatory factor analyses supported structural validity (T1: *χ*^2^/df = 2.82, CFI = 0.90, TLI = 0.91, RMSEA = 0.06; T2: *χ*^2^/df = 3.53, CFI = 0.90, TLI = 0.90, RMSEA = 0.06; T3: *χ*^2^/df = 2.73, CFI = 0.90, TLI = 0.90, RMSEA = 0.05). KMO values supported good sampling adequacy (T1 = 0.81; T2 = 0.84; T3 = 0.88), with significant Bartlett’s tests at all three time points (all *p* < 0.001).

### Analytical strategy

Data were analyzed using SPSS 27.0 and Mplus 8.3. Because all variables were collected via self-report measures, Harman’s single-factor test was conducted to assess common method bias using unrotated exploratory factor analysis across all measurement points (47). Descriptive statistics and Pearson correlation analyses were then performed for all primary variables. To determine whether gender should be included as a covariate in the hypothesized cross-lagged mediation model and whether there were significant changes in the study variable across time, a 3 (time: T1, T2, T3) × 2 (gender: male, female) repeated measures ANOVA was conducted separately for physical activity, prosocial behavior, and psychological resilience. In these analyses, assessment time was the within-subjects factor and gender as the between-subjects factor.

To examine the longitudinal relationships among physical activity, psychological resilience, and prosocial behavior, a structural equation model (SEM) was conducted using Mplus 8.3. A bidirectional cross-lagged mediation model was specified, which simultaneously tested whether (1) physical activity at Time 1 was linked to prosocial behavior at Time 3 via psychological resilience at Time 2, and (2) prosocial behavior at Time 1 was related to physical activity at Time 3 via psychological resilience at Time 2. The model was estimated using the robust maximum likelihood estimator (estimator = MLR). Model fit was evaluated using the chi-square statistic (*χ*^2^), comparative fit index (CFI), Tucker–Lewis index (TLI), root mean square error of approximation (RMSEA), and standardized root mean square residual (SRMR). Comparative model fit was assessed using chi-square difference tests (Δ*χ*^2^/Δdf) to determine the most appropriate model. Indirect effects in the final model were tested using the bias-corrected bootstrap method with 5,000 resamples and 95% confidence intervals. Anonymized data is available online: https://osf.io/my6nf/?view_only=4d8596e464844167a29a4dbf2e8bee36

## Results

### Common method bias test

Twelve factors with eigenvalues greater than 1 were extracted at each time point. The variance explained by the first factor was 19.73% at T1, 23.99% at T2, and 22.61% at T3, all below the recommended 40% threshold, indicating that common method bias was not a major concern in this study.

### Preliminary analysis

Descriptive statistics and Pearson correlations among physical activity, prosocial behavior, and psychological resilience across the three time points are presented in Table 2. All variables were significantly correlated with one another at each time point (*p*s < 0.001), indicating strong concurrent associations. Moreover, significant cross-time correlations were observed between the same constructs measured at different time points, suggesting that physical activity, prosocial behavior, and psychological resilience remained relatively stable over the six-month study period.

**Table 2:** Descriptive statistics and correlations among study variables (*N* = 612).

Variable	*M*	*SD*	1	2	3	4	5	6	7	8
1. Physical activity (T1)	3.22	0.74	1							
2. Prosocial behavior (T1)	3.28	0.67	0.47^***^	1						
3. Psychological resilience (T1)	3.02	0.56	0.48^***^	0.38^***^	1					
4. Physical activity (T2)	3.14	0.71	0.59^***^	0.35^***^	0.39^***^	1				
5. Prosocial behavior (T2)	3.20	0.68	0.49^***^	0.46^***^	0.38^***^	0.59^***^	1			
6. Psychological resilience (T2)	3.06	0.58	0.50^***^	0.44^***^	0.60^***^	0.50^***^	0.57^***^	1		
7. Physical activity (T3)	3.17	0.72	0.34^***^	0.23^***^	0.31^***^	0.47^***^	0.43^***^	0.42^***^	1	
8. Prosocial behavior (T3)	3.23	0.65	0.35^***^	0.24^***^	0.34^***^	0.47^***^	0.46^***^	0.46^***^	0.57^***^	1
9. Psychological resilience (T3)	3.10	0.57	0.29^***^	0.24^***^	0.24^***^	0.34^***^	0.40^***^	0.38^***^	0.39^***^	0.59^***^

^***^p < 0.01.

Physical activity showed a significant change over time, *F*(2, 1,220) = 3.24, *p* = 0.044, *η*^2^ = 0.01, indicating that levels of physical activity varied across the three time points. Follow-up Bonferroni analysis indicated that physical activity at T1 was significantly higher than at T2 and T3. The main effect of gender was not significant, *F*(1, 610) = 0.08, *p* = 0.782, nor was the interaction between time and gender, *F*(2, 1,220) = 2.81, *p* = 0.065. Prosocial behavior also showed significant changes over time, *F*(2, 1,220) = 4.13, *p* = 0.018, *η*^2^ = 0.01. Follow-up Bonferroni analysis revealed that prosocial behavior at T1 was significantly higher than at T2 and T3. The main effect of gender was not significant, *F*(1, 610) = 0.74, *p* = 0.39, nor was the time × gender interaction, *F*(2, 1,220) = 0.59, *p* = 0.549. Similarly, psychological resilience showed a significant main effect of time, *F*(2, 1,220) = 4.51, *p* = 0.014, *η*^2^ = 0.01. Follow-up Bonferroni analysis showed that psychological resilience at T3 was significantly higher than at T1 and T2. The main effect of gender was not significant, *F*(1, 610) = 2.27, *p* = 0.133, and the interaction between time and gender was also not significant, *F*(2, 1,220) = 0.30, *p* = 0.716. Because changes in the study variables were not related to gender, this variable was not included as a covariate in the subsequent longitudinal mediation analyses.

### Longitudinal mediation analysis

**Figure 2:**
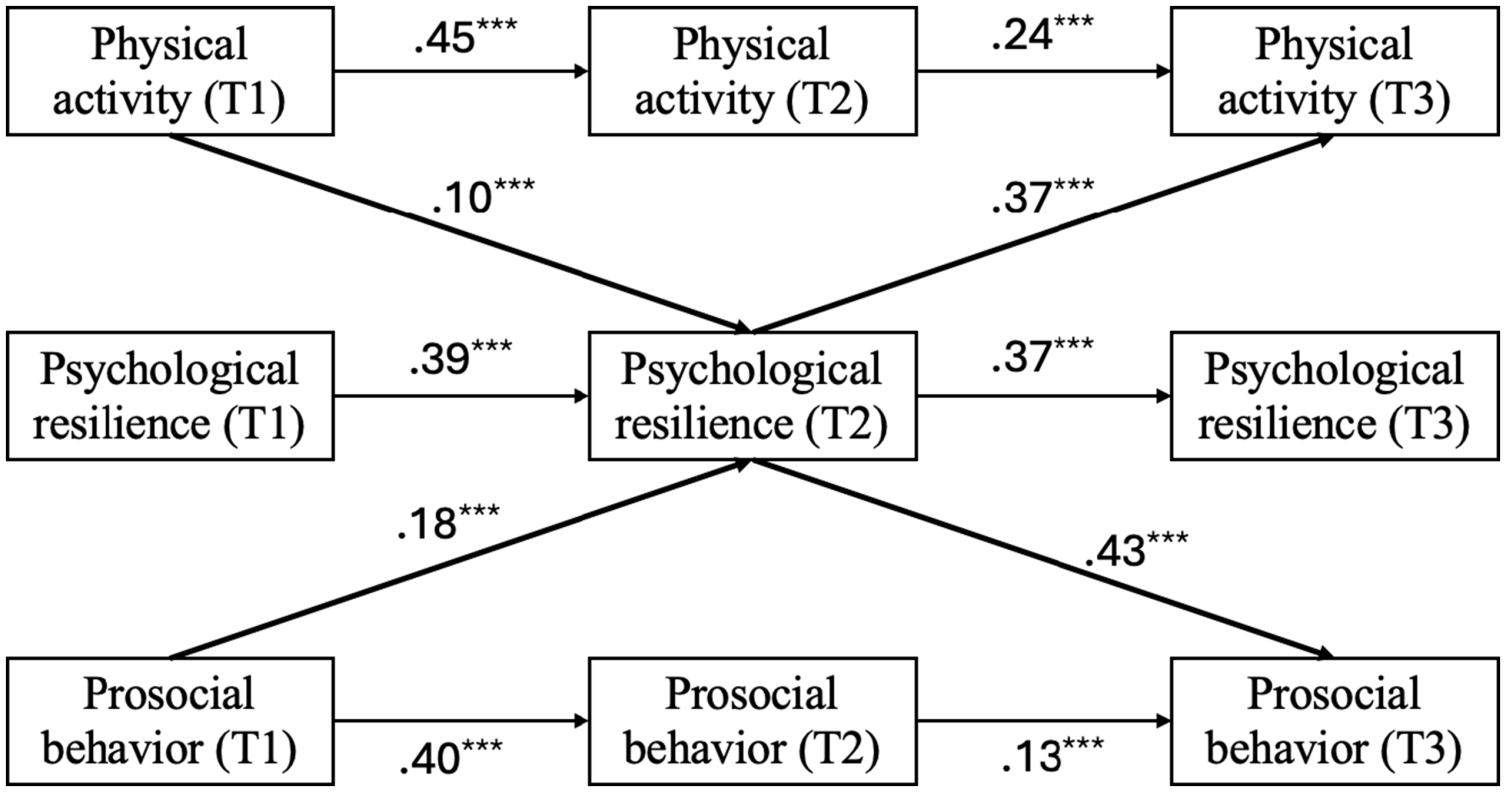
Original study model with standardized coefficients. Thick paths represent primary paths of research interest. For clarity, error terms and covariances are omitted. ^***^p < 0.01.

The original model (Figure 2) exhibited suboptimal fit to the data: *χ*^2^(17) = 200.71, *p* < 0.001; CFI = 0.90; TLI = 0.81; RMSEA = 0.13; SRMR = 0.13. Both hypothesized mediation pathways were statistically supported in this initial model: physical activity at T1 was associated with prosocial behavior at T3 via psychological resilience at T2 (indirect effect = 0.04, SE = 0.02, *p* = 0.01; 95% CI [0.01, 0.08]), and the reverse path from prosocial behavior to physical activity was also significant (indirect effect = 0.07, SE = 0.02, *p* < 0.001; 95% CI [0.04, 0.10]). However, interpretation should be made with caution due to the poor model fit. To improve model specification, three theoretically grounded paths were added based on modification indices: (1) T1 physical activity → T2 prosocial behavior; (2) T2 prosocial behavior → T3 psychological resilience; and (3) T2 physical activity → T3 prosocial behavior.

**Figure 3:**
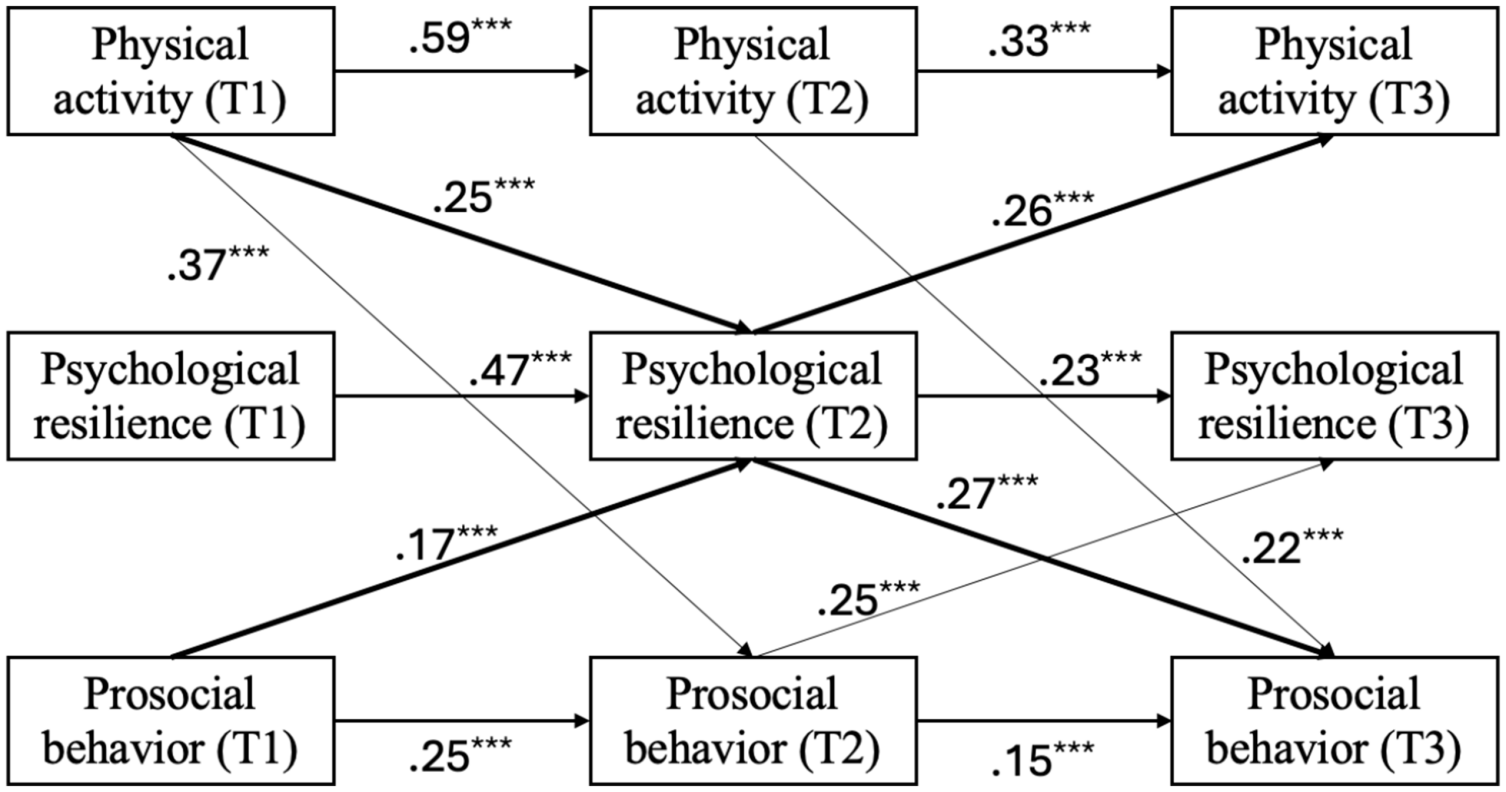
Modified final study model with standardized coefficients. Thick paths represent primary paths of research interest. Thin paths were added based on theoretical rationale and modification indices. For clarity, error terms and covariances are omitted. ^***^p < 0.01.

The revised exploratory model (Figure 3) showed an improved fit: *χ*^2^(14) = 47.28, *p* < 0.001; CFI = 0.98; TLI = 0.94; RMSEA = 0.06; SRMR = 0.04. A chi-square difference test indicated that the revised model provided a significantly better fit than the original model, Δ*χ*^2^(3) = 153.43, *p* < 0.001. Within this final model, both hypothesized mediation pathways were supported. The path from T1 physical activity to T3 prosocial behavior was significant, with psychological resilience at T2 serving as a significant mediator (standardized indirect effect = 0.07, SE = 0.02, *p* < 0.001; 95% CI [0.03, 0.10]). Likewise, the reverse path from T1 prosocial behavior to T3 physical activity was also significant, with psychological resilience again emerging as a significant mediator (standardized indirect effect = 0.04, SE = 0.01, *p* < 0.001; 95% CI [0.02, 0.07]).

Further analyses examined whether specific subdimensions of psychological resilience mediated the longitudinal association between physical activity and prosocial behavior. Neither subdimension showed a significant mediating effect, and thus overall psychological resilience appeared to function as a more integrative construct linking the two. Detailed model information is available in Supplementary Material 1 and Supplementary Table S1.

## Discussion

This three-wave longitudinal study among rural left-behind children identified psychological resilience as a key longitudinal mediator in the bidirectional relationship between physical activity and prosocial behavior. To our knowledge, this is among the first to demonstrate that psychological resilience can not only explain how physical activity is related to prosocial behavior, but also how prosocial behavior, in turn, is associated with sustained physical activity through psychological resilience. These findings suggest that psychological resilience can be a critical developmental bridge between physical and social functioning, highlighting the potential of integrated approaches that simultaneously promote physical activity and prosocial engagement to support psychological well-being in vulnerable populations.

The novel longitudinal evidence for a bidirectional relationship between physical activity and prosocial behavior among rural left-behind children is highlighted. While prior research has primarily emphasized how physical activity is linked to prosocial behavior through mechanisms such as enhanced cooperation, emotional regulation, and group cohesion (17, 22), the current findings contribute to the small but growing literature suggesting that prosocial behavior may also support engagement in physical activity. Extending limited prior research conducted among older adults (24) and other sociocultural context (23, 48), the present findings suggest that left-behind children who exhibit higher levels of prosocial behavior may also be more inclined to engage in physical activity. This idea is similar to past research suggesting prosocial behavior is related to cooperative, group-oriented activities and to seek social connectedness (49) which is beneficial to children’s social adjustment (50). Future research should investigate these mechanisms specific to this population persistence. This reciprocal dynamic underscore the importance of conceptualizing physical and social development as interdependent and mutually reinforcing processes.

Psychological resilience emerged as a key mediating mechanism in the bidirectional relationship between physical activity and prosocial behavior over time. Psychological resilience in this context facilitates the translation of behavioral engagement into socioemotional growth, and vice versa. These findings extend resilience theory (51) by situating resilience within a reciprocal developmental process involving both physical and social domains. Specifically, physical activity may foster resilience by providing structured opportunities for overcoming challenges and promoting positive affectivity, capacities that support prosocial behaviors (36, 52, 53). This is consistent with the neurological evidence suggesting that physical exercise contributes to cognitive improvement and stress resilience in humans and animal models (35). Conversely, prosocial engagement may enhance children’s sense of competence, autonomy, and connectedness (54), thereby strengthening psychological resilience and encouraging future participation in physical activity. This is particularly meaningful for rural left-behind children, whose psychological resilience is often tested by prolonged parental absence and limited social support (55). By identifying resilience as both an outcome and a mechanism of change, the current study underscores its role as a developmental catalyst that amplifies the mutually reinforcing benefits of physical activity and prosocial behavior.

Additionally, physical activity and prosocial behavior were higher at Time 1 than at later waves, whereas psychological resilience increased by Time 3. These temporal patterns may reflect contextual influences such as seasonal variation or changes in academic workload, with greater physical activity and social engagement early in the semester and reduced participation as demands intensified. The later increase in resilience may indicate cumulative adaptation, as participants developed coping skills over time or became more self-aware through repeated assessments. These temporal dynamics likely shaped the mediation process, suggesting that the indirect effect of early physical activity on later prosocial behavior through resilience may vary under different seasonal or contextual conditions.

To improve model fit and better capture the dynamic interplay among variables, three theoretically grounded cross-lagged paths were added: T1 physical activity → T2 prosocial behavior, T2 prosocial behavior → T3 psychological resilience, and T2 physical activity → T3 prosocial behavior. These additions were supported by prior research on reciprocal links between physical and social functioning (17, 23) and the role of social–emotional factors in psychological resilience (54). While theory informed these changes, they were introduced *post hoc* based on modification indices; thus, the final model should be interpreted as exploratory. Replication with pre-registered hypotheses and independent samples is recommended to confirm these pathways.

### Implications

These findings carry important implications for rural left-behind children in China, a population at heightened risk for socioemotional challenges due to prolonged parental separation. Physical activity, when embedded in safe and socially supportive environments, can serve as a low-cost, scalable intervention to promote both psychological resilience and social connectedness. For example, schools could integrate 30 min of cooperative team sports (e.g., basketball or badminton) three times per week into physical education classes, complemented by 15-min weekly prosocial skill-building sessions such as peer tutoring, group problem-solving, or empathy-based discussions. Such combined approaches have been shown to improve social functioning and psychological well-being among children and adolescents (37). Beyond the Chinese context, the results have broader relevance for vulnerable youth globally, including those facing displacement, marginalization, or familial disruption [e.g., sent-away children; (56)]. Theoretically, the demonstrated bidirectional links between physical and social functioning challenge static, linear models of development and call for more dynamic, reciprocal frameworks. Practically, this research supports designing interventions that concurrently promote prosocial engagement, physical activity, and psychological resilience, particularly in under-resourced settings, thereby advancing holistic and sustainable well-being among at-risk youth.

### Limitations

This study is not without limitations. First, the generalizability of the findings is limited. Although participants were recruited from multiple schools, the sample was limited to rural left-behind children in Ji’an City, Jiangxi Province. Given the socioeconomic and educational conditions of the study area, the findings should be generalized to other rural regions with caution, especially to areas with higher economic development or different resource structures. Caution is also warranted when applying these results to international contexts with differing sociocultural and institutional environments. The study adopted a convenience sampling approach, as participating schools were selected based on accessibility and administrative permission, which may have influenced the socioeconomic composition of the sample. Future studies should employ random sampling with broader geographic coverage to enhance representativeness and validate the present findings. Only grade level, rather than participants’ exact ages, was recorded, limiting the precision of developmental analyses. Therefore, these results may not extend to left-behind children from other socioeconomic or regional backgrounds to those living in other countries, or to broader populations of vulnerable youth at different developmental stages without careful cultural and contextual adaptation.

Second, all data were obtained through child self-report, which raises concerns regarding the accuracy of behavioral assessments. For instance, physical activity was measured via self-reported recall, which may be subject to memory errors and imprecise estimation of the actual frequency and intensity of engagement. The absence of parent, teacher, or peer reports may affect the validity of behavioral assessments, particularly for prosocial behavior. However, this approach was chosen for its feasibility in large-scale, school-based multiwave data collection. Likewise, prosocial behavior is vulnerable to social desirability bias, with children potentially overreporting helpful or cooperative actions. Future research would benefit from incorporating more objective assessments, such as accelerometers to quantify physical activity or teacher and peer reports to corroborate prosocial behavior ratings, to improve measurement validity. When available, sensitivity analyses comparing self-reported physical activity with school physical education attendance records could further assess self-report bias.

Third, while the three-wave design over a six-month period allows for valuable longitudinal insights, future studies with longer follow-up periods are needed to capture more stable developmental trajectories and enduring effects. Fourth, while the model supports bidirectional associations over time, these findings should be interpreted with caution, as statistical reciprocity in SEM does not imply true bidirectional causality. Also, it should be noted that the final model, developed based on modification indices, is exploratory in nature. To verify its robustness and mitigate overfitting risks, future research could adopt pre-registered designs and cross-validation approaches to test the added paths in independent samples. Experimental or intervention-based studies are necessary to confirm the directional mechanisms identified. Finally, although the model offers a strong theoretical foundation for exploring psychological resilience as a dynamic mechanism within developmental cascades, it assumes that psychological resilience functions symmetrically across behavioral and social domains, which may oversimplify the nuanced roles psychological resilience plays in different contexts.

### Future directions

Building on the current findings, several avenues warrant further investigation. First, future research should develop and evaluate school- or community-based interventions that combine physical activity with prosocial skill training to enhance psychological resilience among left-behind and other marginalized children. Embedding resilience-building activities within physical education or peer mentoring programs could help establish causal links and inform practical applications. Second, longitudinal studies with longer follow-up periods are needed to assess the stability of the observed developmental pathways across time and contexts. Replicating this model in different regions of China and among diverse youth populations (e.g., migrant or urban low-income children) would clarify the cultural and contextual generalizability of the findings. Third, incorporating multi-informant approaches (e.g., parent, teacher, or peer reports) and objective indicators, such as accelerometers for physical activity or observational assessments of prosocial behavior, would enhance measurement validity. Lastly, examining moderating factors, such as caregiving arrangements, school support, or socioeconomic context, as well as testing additional mediators (e.g., emotion regulation, peer attachment, self-efficacy), could deepen understanding of the mechanisms linking physical activity, psychological resilience, and prosocial behavior.

## Conclusion

This study provides novel longitudinal evidence of a bidirectional relationship between physical activity and prosocial behavior, with psychological resilience serving as a key mediating mechanism among rural left-behind children in China. By identifying psychological resilience as both a pathway and a product of behavioral and social engagement, the findings advance theoretical models of child development and highlight critical actionable points for intervention. Promoting physical activity and prosocial engagement simultaneously may offer a scalable, low-cost strategy to support the well-being of structurally disadvantaged youth. Future research should build on this foundation to design contextually sensitive, developmentally appropriate, and mechanism-informed programs that foster holistic development across diverse populations.

## Data Availability

The datasets presented in this study can be found in online repositories. The names of the repository/repositories and accession number(s) can be found below: https://osf.io/my6nf/?view_only=4d8596e464844167a29a4dbf2e8bee36.
