# Supplementary material for: The reciprocal relationship between physical activity and prosocial behavior among rural left-behind children: a cross-lagged mediation analysis of psychological resilience
Source: Front Public Health. 2025 Dec 3;13:1690603. doi: 10.3389/fpubh.2025.1690603 (PMC12708260; doi:10.3389/fpubh.2025.1690603)
Supplement: Supplementary file 1 [file Supplementary_file_1.docx]

**Supplementary Material 1**

Additional cross-lagged mediation analyses were conducted to test whether specific subdimensions of psychological resilience mediated the longitudinal relationship between physical activity and prosocial behavior. Based on Hu and Gan’s (2008) framework, two subdimensions of psychological resilience were used: Personal Strength (measured using goal focus, emotional control, positive cognition subscales) and Support Strength (measured family support and interpersonal assistance subscales). Thus, two cross-lagged models were tested for the two psychological resilience subdimensions (i.e., Personal Strength and Support Strength).

**The Cross-Lagged Mediation Analysis with the Personal Strength Dimension of Psychological Resilience**

The initial cross-lagged model for the Personal Strength dimension showed unsatisfactory model fit (χ²/df = 8.61, CFI = 0.852, TLI = 0.712, RMSEA = 0.158, SRMR = 0.116), indicating that the original model specification required optimization. To improve model quality, systematic modifications were conducted based on the modification indices while ensuring theoretical rationality. The main adjustments included: allowing correlations among measurement errors of the same observed variable across different time points, and adding key theoretically justified paths, including the direct path from T1 physical activity to T2 prosocial behavior, the cross-period stability path from T1 to T3 Personal Strength, and the reverse path from T2 prosocial behavior to T3 physical activity, to more comprehensively reflect the longitudinal and bidirectional relationships among variables.

After these modifications, model fit indices improved significantly and reached a good level (χ²/df = 1.94, CFI = 0.985, TLI = 0.964, RMSEA = 0.055, SRMR = 0.036; Table S1), indicating satisfactory model fit. Based on the final model, path analysis results showed that T1 physical activity had a significant total effect on T3 prosocial behavior (*β* = 0.170, *p* < 0.001), and this effect was fully mediated through indirect paths. However, the specific indirect path via T2 Personal Strength was not significant (*β* = 0.000, *p* = 0.795), suggesting that Personal Strength did not play a longitudinal mediating role between physical activity and prosocial behavior. In contrast, the indirect path via T2 prosocial behavior was significant (*β* = 0.170, *p* < .001), indicating that prosocial behavior itself had cross-time stability. In addition, the study also identified a reverse path effect, whereby T1 prosocial behavior significantly predicted T3 physical activity through T2 prosocial behavior (*β* = 0.092, *p* < 0.001), suggesting a bidirectional facilitative relationship between the two variables.

**The Cross-Lagged Mediation Analysis with the Support Strength Dimension of Psychological Resilience**

The initial cross-lagged model for the Support Strength dimension also showed poor model fit (χ²/df = 4.58, CFI = 0.860, TLI = 0.799, RMSEA = 0.108, SRMR = 0.112). To improve model quality, systematic modifications were made following the successful adjustment approach used for the Personal Strength model.

After modification, the model fit improved substantially (χ²/df = 1.33, CFI = 0.989, TLI = 0.981, RMSEA = 0.033, SRMR = 0.037; Table S1), providing a reliable basis for analyzing mediation effects in this dimension. Path analysis results based on the Support Strength model indicated that there was a significant total effect of physical activity on prosocial behavior over time, but this process was not mediated through the Support Strength dimension. Specifically, T1 physical activity had a significant total effect on T3 prosocial behavior (*β* = 0.171, *p* < .001), and this effect was fully mediated through indirect paths. The specific indirect path through T2 Support Strength was not significant (*β* = 0.000, *p* = .968), whereas the indirect path through T2 prosocial behavior was significant (*β* = 0.171, *p* < .001). At the same time, a significant reverse path effect was observed: T1 prosocial behavior positively predicted T3 physical activity through T2 prosocial behavior (*β* = 0.091, *p* < 0.001). These results indicate that in the longitudinal relationship between physical activity and prosocial behavior, Support Strength did not serve as a mediator. Instead, the stability of prosocial behavior itself was the key mechanism, and the two constructs demonstrated a bidirectional facilitative relationship.

Reference:

Hu, Y. Q., & Gan, Y. Q. (2008). Development and psychometric validity of the resilience scale for Chinese adolescents. *Acta Psychologica Sinica*.

| Table S1. **Fit Indices and Path Coefficients for Cross-Lagged Models of Psychological Resilience Subdimensions** | | | | | | | |
| --- | --- | --- | --- | --- | --- | --- | --- |
| Model & Path | χ²/df | CFI | TLI | RMSEA | SRMR | Total Effect (T1 Physical Activity → T3 Prosocial Behavior) | Indirect Effect (T1 Physical Activity →[mediator]→ T3 Prosocial Behavior) |
| **Personal Strength Model** | 1.94 | 0.985 | 0.964 | 0.055 | 0.036 | 0.170^***^ |  |
| T1 Physical Activity → T2 Personal Strength → T3 Prosocial Behavior |  |  |  |  |  |  | 0.000 |
| T1 Physical Activity → T2 Prosocial Behavior → T3 Prosocial Behavior |  |  |  |  |  |  | 0.170^***^ |
| **Support Strength Model** | 1.33 | 0.989 | 0.981 | 0.033 | 0.037 | 0.171^***^ |  |
| T1 Physical Activity → T2 Support Strength → T3 Prosocial Behavior |  |  |  |  |  |  | 0.000 |
| T1 Physical Activity → T2 Prosocial Behavior → T3 Prosocial Behavior |  |  |  |  |  |  | 0.171^***^ |
| Note: ^*^p < .05, ^**^p < .01, ^***^p < .001 | | | | | | | |
